# Cell Junction and Vesicle Trafficking-Mediated Melanosome/Melanin Transfer Are Involved in the Dynamic Transformation of Goldfish *Carassius auratus* Skin Color

**DOI:** 10.3390/ijms232012214

**Published:** 2022-10-13

**Authors:** Lili Liu, Xiaowen Wang, Rong Zhang, Huijuan Li, Hua Zhu

**Affiliations:** 1 Fisheries Research Institute, Beijing Academy of Agriculture and Forestry Sciences, Beijing 100068, China; 2 Beijing Key Laboratory of Fishery Biotechnology, Beijing 100068, China

**Keywords:** goldfish, skin color transformation, melanophore, cell junction, vesicle trafficking

## Abstract

Goldfish are one of the most popular models for studying the genetic diversity of skin color. Transcriptome sequencing (RNA-seq) and whole genome bisulfate sequencing (WGBS) of skin tissues from the third filial (F3) cyan (CN), black (BK), and white (WH) goldfish were conducted to analyze the molecular mechanism of color transformation in fish. The RNA-seq yielded 56 Gb of clean data and 56,627 transcripts from nine skin samples. The DEGs (differentially expressed genes) were enriched in cell junction cellular components and the tight junction pathway. Ninety-five homologs of the claudin family were predicted and 16 claudins were identified in correlation with skin color transformation. WGBS yielded 1079 Gb of clean data from 15 samples. Both the DEGs and the DMRs (differentially methylated regions) in the BK_CN group were found to be enriched in cytoskeleton reorganization and vesicle trafficking. Masson staining and TEM (transmission electron microscopy) confirmed the varied distribution and processes of melanosome/melanin in skin tissues. Our results suggested that cytoskeleton reorganization, cell junction, and the vesicle trafficking system played key roles in the transfer of the melanosome/melanin, and it was the extracellular translocation rather than the biosynthesis or metabolism of the melanin process that resulted in the color transformation of cyan goldfish. The data will facilitate the understanding of the molecular mechanisms underlying dynamic skin color transformation in goldfish.

## 1. Introduction

Goldfish (*Carassius auratus*) are one of the world’s most beloved aquarium mainstays [1]. This teleost fish has been subjected to extremely intensive artificial selection in China over the past 1000 years [2]. Goldfish first arrived in Europe and Japan in the 1600s, followed by the United States in the 1800s, and have become what is likely the first foreign fish species introduced to North America (https://www.cabi.org/isc/datasheet/90563#tosummaryOfInvasiveness (accessed on 19 August 2022)).The ancestors of present-day goldfish underwent a fourth round of whole genome duplication (WGD) millions of years ago in addition to the teleost-specific WGD, contributing to the pigment diversification and complexity of pigmentation gene regulation [3,4]. After the breeding and mutations enforced by its domestication history, there are currently dozens of types of goldfish breeds with various colors, ranging from black to orange, red, and white.

As a result of the release of a high-quality reference genome [4] and large-scale whole genome/transcriptome sequencing data [5,6] for goldfish, the molecular mechanisms underlying the variations in goldfish coloration have been elucidated. Cyan goldfish, with their immensely imaginative “ching” color, are considered probably the best reflection of traditional Chinese aesthetic characteristics. With its great instability, the morphological color of cyan (CN) goldfish often transforms to white (WH) or black (BK). This morphological color change occurs within days and/or weeks and involves variations in pigment concentration or the density and distribution of the chromatophores in the skin [7]. Intensive works have been conducted to elucidate the genomic network of skin pigmentation in fish in recent years [8]. For example, genes including *pax7*, *mitf*, *tyr*, and *tyrp1* were identified in association with early skin pigmentation [9], while the Wnt/bcatenin signaling pathway might be regulating the depigmentation [10]. MicroRNAs are also reported to have vital roles in regulating the skin pigmentation [11]. DNA methylation sites, such as *mitfa*, *tyr*, *dct*, *foxd3*, and *hpda*, were observed in the signaling pathways, including MAPK, cAMP, endocytosis, melanogenesis, and Hippo [12]. However, the molecular mechanisms of the goldfish dynamic morphological color transformation remain unclear. We therefore constructed a hybrid family using red grass goldfish and cyan wakin goldfish to study the dynamic transformation mechanism of goldfish skin color.

## 2. Results

### 2.1. Summary of Transcriptome Sequencing Data

**Figure 1:**
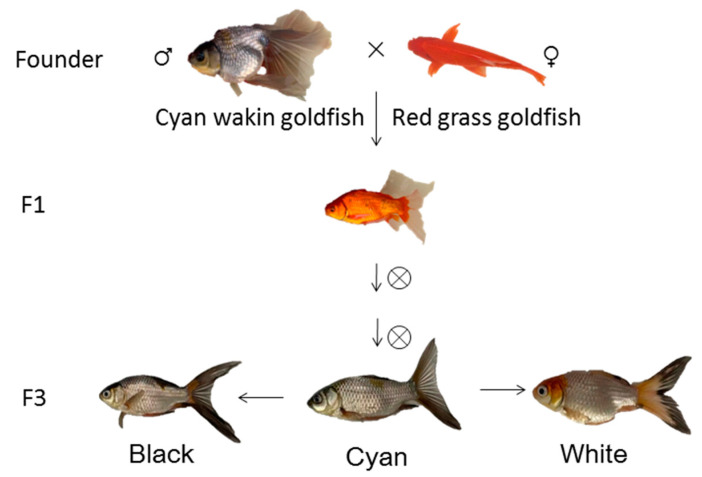
The construction flow of hybrid goldfish family. F1: the first filial generation; F3: the third filial generation. ♂: male; ♀: female; ×: cross breeding; ⊗: self-cross breeding.

**Figure 2:**
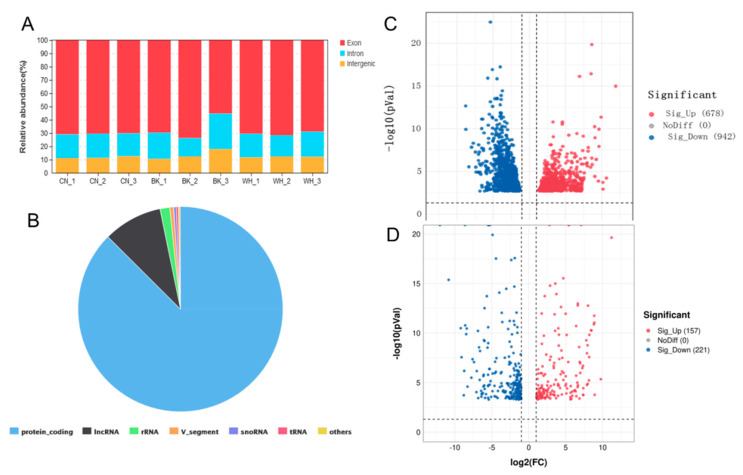
Summary of transcriptome sequencing data. (**A**) The biotypes of transcripts in goldfish. (**B**) Mapping percentage of uniquely mapped reads. (**C**) Differentially expressed genes in black_cyan goldfish group. (**D**) Differentially expressed genes in white_cyan goldfish group.

To identify the genes involved in goldfish color transformation, we quantified the gene expression differences between three fish with CN, BK, and WH skin, with red grass goldfish and cyan wakin goldfish as parents (Figure 1). Less than 0.5% of the second filial (F2) generation had cyan skin, and the rest had fully red skin. In the third filial (F3) generation, the skin colors of most of the fish were cyan with red spots; the rest still had fully red skin. RNA sequencing yielded a total of 56 Gb of clean data from nine individual skin samples. The replicates for each tissue sample were sequenced separately, and the average number of reads per sample was 41,758,454.2. The reads from each replicate were mapped to the reference genome for *Carassius auratus* (assembly ASM336829v1), with an average mapping rate of 87.94% (Supplementary Table S1). Uniquely mapped reads were mapped mainly to the exons (55.24~73.74%), introns (13.84~26.79%), and intergenic regions (10.57~17.97%) (Figure 2A). A total of 56,627 transcripts were identified in the goldfish, including 49,525 transcripts from protein coding genes, 5279 from long noncoding RNAs (lncRNAs), 873 from rRNA genes, 217 from tRNA genes, and 732 other transcripts, such as small nucleolar RNAs (snoRNAs), small nuclear RNAs (snRNAs), transcribed_pseudogenes, guide RNA, constant regions (C regions) and variable chains (V segments) (Figure 2B).

There were 1620 differentially expressed genes (DEGs) identified from the BK_CN group based on the RNA-seq analysis, including 678 up-regulated genes and 942 down-regulated genes (Figure 2C). In the WH_CN group, 378 DEGs were identified including 157 up-regulated genes and 221 down-regulated genes (Figure 2D). The BK_CN and WH_CN groups shared 240 DEGs, including 137 up-regulated genes and 103 down-regulated genes.

### 2.2. Summary of Whole Genome Bisulfite Sequencing (WGBS) Analysis

In order to analyze the genomic cytosine methylation pattern, we quantified the methylation rates from skin samples of cyan, black, and white goldfish using five replicates each. The WGBS analysis resulted in 1079 Gb of clean data. The clean reads from 15 samples ranged from 409,370,890 to 517,243,373 reads per sample. The average Q30 after filtering was 90.63%. The reads from each sample were mapped to the reference goldfish genome with an average rate of 69.85%. The unique mapped rate ranged from 59.68 to 60.86%. The total number of cytosine (C) bases in the whole genome ranged from 519,819,334 to 533,225,010. The average ratios of the C bases in the context of CGs, CHGs, and CHHs were 9.14%, 21.28%, and 69.38%, respectively (Supplementary Table S2).

Three types of methylation DNA sites were found in this dataset, including methylated CGs (mCG), methylated CHGs (mCHG), and methylated CHHs (mCHH) in goldfish. The total number of methylated cytosine (mC) sites in each sample was 23,425,406–27,214,840. Of these, the mCG sites accounted for 98.98–99.04%, followed by the mCHH and mCHG sites. The average number of mCG, mCHG, and mCHH sites was 24,965,893, 56,752, and 192,196, respectively. The number of methylation sites in the mCG context was the most prevalent, while mCHGs and mCHHs accounted for an extremely low proportion of methylated CGs (Supplementary Table S3). The level rank of mCG in genomic regions was last exon > internal intron > internal exon > first intron > downstream > upstream > first exon. The findings suggested that the methylation level in the gene regions was much higher than in the intergenic regions or promoter regions. The first exon regions maintained a very low methylation level (Supplementary Figure S1). The Pearson correlation coefficient of all the samples was generally higher than 0.88, suggesting that the methylation patterns were similar across all samples.

A total of 1926 differentially methylated regions (DMRs) were identified in the BK_CN group, including 1032 hyper DMRs and 894 hypo DMRs. Most of these DMRs were located in the intron, exon, and promoter regions, accounting for 949, 400, and 340, respectively. A few DMRs were located in the 5′UTR, CpG island, and 3′UTR regions. There were 1090 DMRs (611 hyper and 479 hypo) in the WH_CN group, sharing the same tendency of distribution for the functional regions (Supplementary Figure S2).

### 2.3. Cell Junction-Related Pathways and Functions Were Enriched in DEGs and DMRs in Goldfish

**Figure 3:**
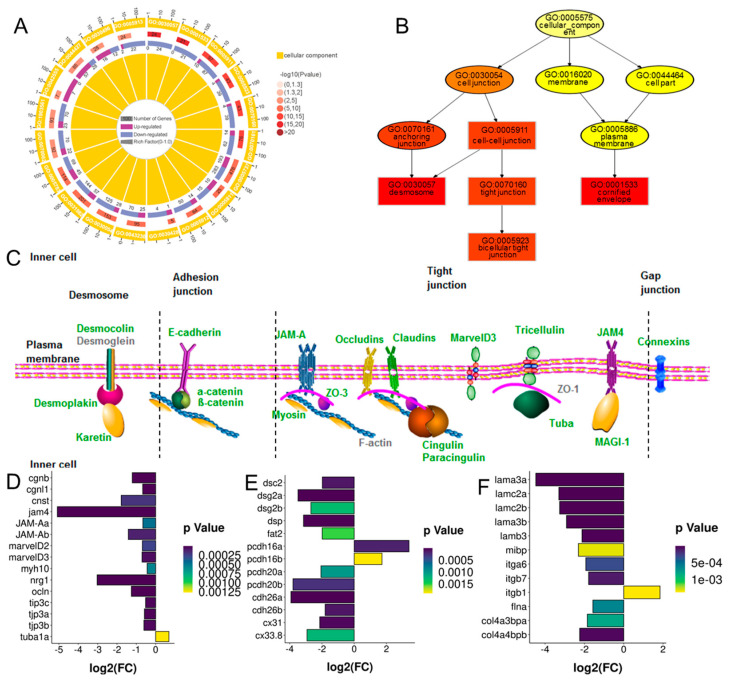
The enrichment of differentially expressed genes (DEGs) in black_cyan group. (**A**) GO enrichment of up- and down-regulated DEGs in terms of cellular component. (**B**) Directed acyclic graph of top enriched cell junction terms. (**C**) Transmembrane proteins in cell junctions. (**D**–**F**) The expression of DEGs involved in cell junctions in black_cyan group.

In the BK_CN group, the 1620 DEGs consisted of 1576 protein-coding genes, 40 lncRNAs, and other types of genes. The differently expressed coding genes were enriched in cell junction components such as cell–cell junction (GO: 0005911), tight junction (GO: 0070160), bicellular tight junction (GO: 0005923), desmosome (GO: 0030057), and cornified envelope (GO: 0001533), based on Gene Ontology (GO) enrichment analysis. The majority of the DEGs in the above terms displayed a decreasing expression pattern (Figure 3A,B). The DEGs were enriched in a tight junction pathway based on KEGG enrichment (padj < 0.005). The expression of the genes in the tight junction pathway was down-regulated significantly and included occludin (*ocln*), JAM4 (*jam4*), JAM-A (*jam-Aa* and *jam-Ab*), marvelD3, tricellulin (*marvelD2*), ZO-3 (*tjp3a*, *tjp3b* and *tjp3c*), tubulin α-1A (*tuba1a*), cingulin (*cgnb*), and paracingulin (*cgnl1*) (Figure 3C,D). The expression of the genes involved in the adhesion junctions, desmosomes, and gap junctions shared similar expression tendencies, including E-cadherin (*cad26a* and *cad26b*), α-catenin and β-catenin, desmocollin 2 (*dsc2*), desmoglein (*dsg2a* and *dsg2b*), and connexin (*cx31* and *cx33.8*) (Figure 3C,E). The expression of the genes involved in cell–matrix adhesion, such as integrin (*itga6b* and *itgb7)*, muscle-specific beta 1 integrin binding protein *(mibp)*, filamin *(fln)*, collagen IV *(col4)*, and laminin *(lama3a*, *lama3b*, *lamb*, *lamc2a* and *lamc2b)* decreased significantly as well (Figure 3F). These data suggested that the DEGs in the BK_CN group were enriched in the cell–cell junction and cell–matrix junction proteins and had a predominant pattern of decreased expression.

**Figure 4:**
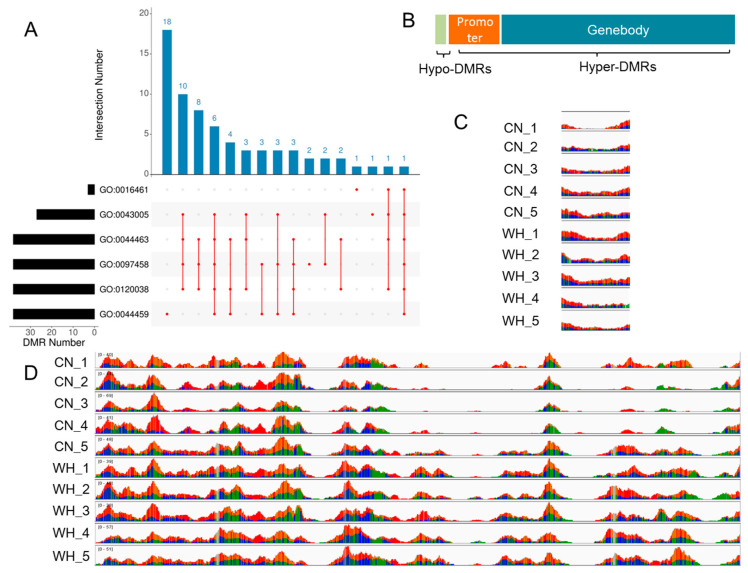
Differentially methylated regions (DMRs) analysis in white_cyan (WH_CN) group. (**A**) The Gene Ontology enrichment of DMRs. (**B**) Gene function analysis of DMRs. (**C**) Methylation coverage of *claudin 22* gene in Cn and WH goldfish. (**D**) Methylation coverage of protocadherin gene in Cn and WH goldfish.

The DMRs in the WH_CN group were then annotated and were found to be mainly enriched in cell membrane components, including the plasma membrane part (GO: 0044459), neuron part (GO: 0097458), cell projection part (GO: 0044463), plasma membrane bounded cell projection part (GO: 0120038), and neuron projection (GO: 0043005) (Figure 4A). Notably, 96% of the DMRs in the above terms were found to have hypermethylation levels in white goldfish; 77% of these hyper-DMRs were found in the gene body regions and 18% in the promoter regions (Figure 4B). Hyper-DMRs in the WH_CN group were identified in genes mainly associated with the cell junction, such as claudin 22 (Figure 4C) and protocadherin (Figure 4D), suggesting the possibility of gene expression decrease in white goldfish.

**Figure 5:**
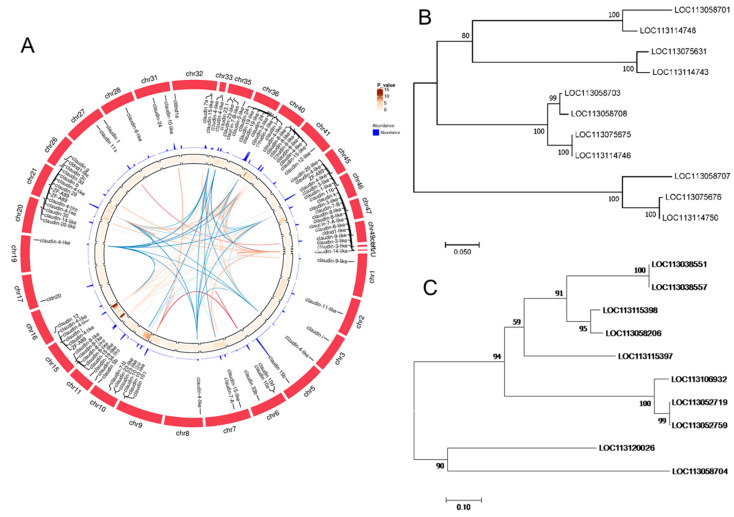
The claudin genes family in goldfish based on RNA-seq. (**A**) The expression, chromosome location, and link of claudins in goldfish skin tissues. (**B**) The molecular phylogenetic analysis of claudin 8-like genes by maximum likelihood method. (**C**) The molecular phylogenetic analysis of claudin 4-like genes by maximum likelihood method.

A total of 95 gene members of the claudin family in goldfish were predicted based on our RNA sequencing data, including homologous genes for claudins 1, 3,3-like, 4-like, 5-like, 6-like, 7, 8, 8-like, 9-like, 10, 11, 12, 15, 19, 20, 20-like, 22, 23, 24, 29, 30, 33, b, f, g, i, j, k, and claudin domain-containing protein 1 (*cldnd1*). These genes were located on 31 chromosomes and 11 unplaced scaffolds and on chr15, chr21, and chr40 in particular. The claudin 4-like genes shared 16 homologous genes located on eight chromosomes. The claudin 8-like genes shared 11 homologous genes located on two chromosomes and three scaffolds. Sixteen claudins in the BK_CN group were identified to have significantly decreased expression (padj < 0.05) (Figure 5A). In the present study, both the claudin 8-like (Figure 5B) and the claudin 4-like genes (Figure 5C) were clustered into three clades based on molecular phylogenetic analysis, suggesting that the gene expansion might be a result of genome duplication events.

### 2.4. Differential Expression and Methylation of Cytoskeleton Organization and Vesicle Trafficking Component-Related Genes

**Figure 6:**
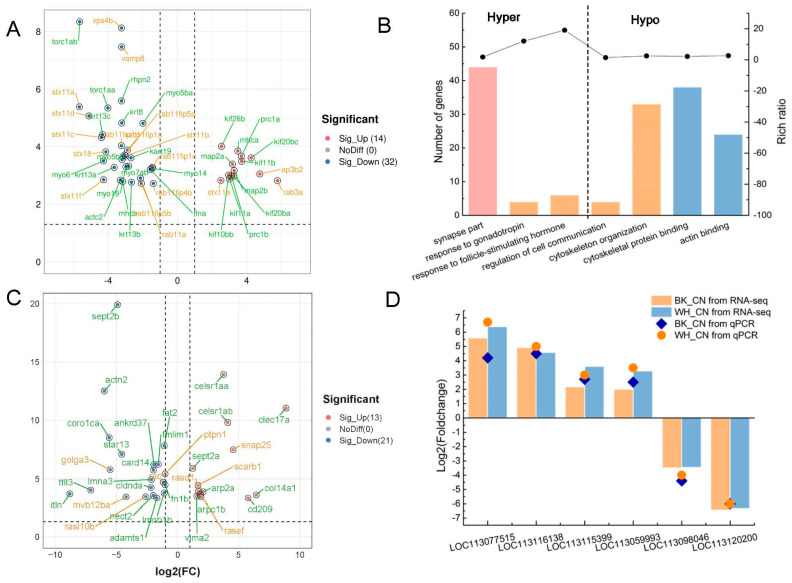
The differentially expressed genes (DEGs) and differentially methylated regions (DMRs) related with cytoskeleton organization and vesicle trafficking. (**A**) DEGs related with cytoskeleton organization and vesicle trafficking in black_cyan group. (**B**) GO enrichment of DMRs in black_cyan group. (**C**) DEGs related with cytoskeleton organization and vesicle trafficking in white_cyan group. (**D**) qPCR verification of DGEs in black_cyan and white_cyan group.

A panel of DEGs associated with cytoskeleton organization in the BK_CN group were subsequently identified, including myosin (*myo5ba*, *myo5bb*, *myo6*, *myo7ab*, *myo14* and *myo19*); microtubule-associated protein 2 (*map2a* and *map2b*); Ras-related protein Rab-11A (*rab11a*); Rab11 family-interacting protein (*rab11fip1a*, *rab11fip11b*, *rab11fip11c*, *rab11fip4b*, *rab11fip5a* and *rab11fip5b*); kinesin-like protein (*kif11a*, *kif11b*, *kif20ba*, *kif20bb*, *kif20bc* and *kif26b*); protein regulator of cytokinesis 1 (*prc1a* and *prc1b*); and torsin-1A (*tor1aa* and *tor1ab*) (Figure 6A). Similarly, the DMRs in the BK_CN group were enriched in cytoskeleton organization-related biological processes, including cytoskeletal protein binding and actin binding functions (Figure 6B). 

A total of 378 DEGs in the WH_CN group were also identified and enriched predominantly in the cytoplasm (GO: 0005737), perinuclear region of cytoplasm (GO: 0048471), laminin complex (GO: 0043256), and vacuole-mitochondrion membrane contact site (GO: 1990816) cellular components. The great majority of cytoskeleton organization-related genes exhibited decreased expression, such as cadherin EGF LAG seven-pass G-type receptor 1-like (*celsr1aa* and *celsr1ab*); coronin-1C-like (*coro1ca*); actin family protein 2-B-like (*arp2a*); scavenger receptor class B member 1 (*scarb1*); tubulin tyrosine ligase-like family, member 3 (*ttll3*); vimentin A2-like (*vima2*); septin-2 (*sept2a* and *sept2b*); fibronectin 1b (*fn1b*); and protocadherin Fat 2-like (*fat2*) (Figure 6C). 

Meanwhile, the expression levels of the vesicular transport-related genes changed when the goldfish colors transformed from cyan to black and/or white. For example, in the black goldfish, the expression of syntaxin-11 (*stx11a*, *stx11b*, *stx11c*, *stx11d* and *stx11f*); stx18, vesicle-associated membrane protein 8 (*vamp8*); vacuolar protein sorting-associated protein 4B (*vps4b*) and Rab11 family-interacting protein 1 (*rab11fip1a*, *rab11fip1b* and *rab11fip1c*); Rab11 family-interacting protein 4 (*rab11fip4b*); and Rab11 family-interacting protein 5 (*rab11fip5a* and *rab11fip5b*) decreased significantly (pval < 0.005) (Figure 6A). Similarly in the white goldfish, the expression of multivesicular body subunit 12B (*mvb12ba*) and synaptosomal-associated protein of 25 kDa (*snap25*) changed significantly (Figure 6C). The relative expression of the DEGs associated with the cytoskeleton identified in both the BK_CN and WH_CN groups was next verified by the real-time quantitative PCR (qPCR) method and included *sept2b* (LOC113116138) and myosin heavy chain and fast skeletal muscle (*myh*, LOC113098046). The qPCR results showed a similar relative expression trend with the RNA-seq data, suggesting the data reliability of the transcriptomics profilings (Figure 6D).

### 2.5. Masson Trichrome Staining and Transmission Electron Microscopy (TEM)

**Figure 7:**
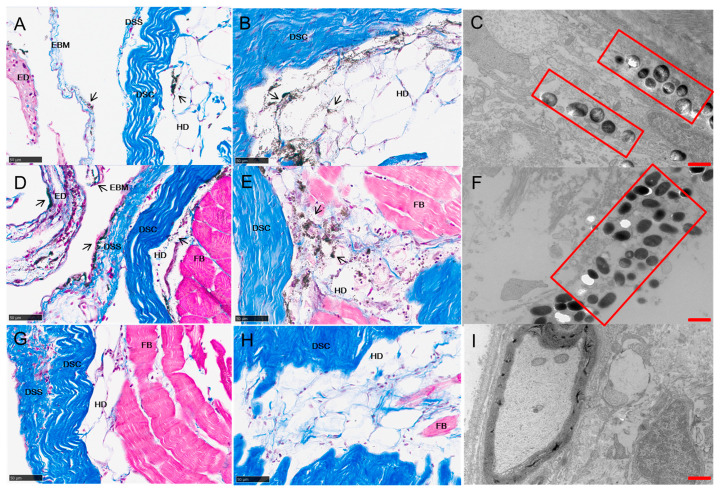
The location and structure of melanophores and melanosomes in skin tissues of cyan (**A**–**C**), black (**D**–**F**), and white (**G**–**I**) goldfish. Masson trichrome staining sections (**A**–**H**) suggested the differential strategy of location in goldfish skin layers. Transmission electron microscopy suggested the structure difference of melanosomes. ED: epidermal, EBM: epidermal basement membrane, DSS: dermal stratum spongiosum, DSC: dermal stratum compactrum, HD: hypodermal, FB: fibroblast. Black arrow indicates melanophores. Red rectangle box indicates melanosomes. Black scale bar: 50 μm; Red scale bar: 10 μm.

Skin tissue sections from the cyan, white, and black goldfish were stained by Masson trichrome for visualization. Dispersed melanophores were observed below the epidermal basement membrane (EBM), the dermal stratum spongiosum (DSS), and in the hypodermis (HD) of the cyan goldfish (Figure 7A). The HD contained multiple layers of large connective cells filled with lipids. Melanophores with irregular dendrite protrusions were found to extend into the intercellular space between the adipocytes and fibroblasts, parallel to the HD layer (Figure 7B). The melanophores contained different sizes of pigmented organelles (melanosomes). Most of the organelles were lacking in melanin granules, suggesting that the melanosomes were immature and at an early stage of the melanosome development process (Figure 7C). In the black goldfish, a continuous melanosome layer was seen below the EBM, as well as a thinner melanosome layer in the HD. Apparently different to cyan goldfish, certain amounts of dispersed melanosomes were observed in not only the DSS, but also in the epidermis, as well as the connectivity layer between the DSS and the dermal stratum compactum (DSC). The melanophores were full of melanosomes and large in size (Figure 7D,E); however, the melanosomes varied little in size and density. Individual melanosomes lost pigment granules completely, leaving cavities (Figure 7F). Neither melanophores (Figure 7G,H) nor melanosomes (Figure 7I) were observed in the skin tissues of the white goldfish, based on Masson trichrome staining and TEM microscopy. However, the amount of nucleolus seemed unequal to the amount of adipocyte cells in the HD, suggesting the possibility of the existence of melanophores without melanosomes (Figure 7H).

## 3. Discussion

The present study elucidated the molecular mechanism underlying the dynamic transformation of goldfish skin color from cyan to black and/or white by RNA sequencing and WGBS. qPCR was utilized to determine the differential expression level. The expression profiling of most of these DEGs was in agreement with the results of our transcriptome analysis, indicating that there was no consistent bias in the expression profile. The results from the present study indicated that DEGs and DMRs with a possible role in goldfish dynamic color transformation could be classified into two major groups: (1) genes involved in cell junctions, especially those coding for proteins involved in tight junctions and (2) genes with a molecular function in cytoskeleton organization and vesicle trafficking. Melanin biosynthesis or its regulation/metabolism process in pigmentation has been reported [8]. t is noteworthy that none of melanin biosynthesis, its regulation/metabolism process and melanophore development/differentiation-related pathways were enriched in our study.

### 3.1. Cell Junctions Involved in the Dynamic Transformation of Goldfish Skin Color

The roles for the cell–cell and cell–matrix interactions in the pigment cell development have been suggested by classical experiments and more recent observations [13,14]. Pigment cells from some species develop persistent contacts with one another [15] or express different cell–cell adhesion molecules as they mature [16]. The maintenance of different pigment patterns in marble trout (labyrinthine skin pattern) and brown trout (spotted skin pattern) has been found to be dependent upon chromatophores communication, involving gap junctions, tight junctions, and ion channels [17]. Unigenes from eight Sinocyclocheilus cavefishes with different skin colors have been mapped to focal adhesion, regulation of actin cytoskeleton, and calcium signaling pathways [18]. 

In the present study, the DEGs in the BK_CN group were annotated and mapped to cell–cell junction genes and cell–matrix junction genes. Meanwhile, the DMRs in the WH_CN group were annotated and mapped to the plasma membrane components and cell projection components, suggesting that cell junctions, especially tight junctions, were involved in the dynamic transformation of goldfish skin color from cyan to black and/or white. In the black goldfish, the expressions of all the transmembrane proteins involved in the tight junctions were down-regulated, including the claudins, occludins, tricellulin, marvelID3, JAM-A, and JAM4. The transmembrane proteins that participated in the adhesion junctions, gap junctions, and desmosomes showed a similar expression pattern. Thus, the connectivity between the melanophores and the surrounding cells reduced on all fronts. The melanosome/melanin was able to transfer out of the melanophores, contributing to the translocation of the pigment granules. Our results suggested that cell junction was involved in the dynamic transformation of adult goldfish skin color.

Claudins are a family of tetraspanins involved in the formation of tight junction strands [19]. Claudin and claudin-like genes have been reported from lower chordates to mammals. There are 16 claudins in the sea lamprey (*Petromyzon marinus*) [19] and 23 claudins in the frog (*Xenopus tropicalis*) [20]. At least 27 claudin members in mammals have been reported to date [21]. Teleosts are particularly rich in claudin homologues. Fifty-four claudin genes have been found in zebrafish (*Danio rerio*) [20] and 56 claudins in puffer fish (*Fugu rubripes*) [22], due to gene duplication processes rather than the whole genome duplication of the extent of the teleost fish [22,23]. At least 94 claudins have been identified as being expressed to some extent in goldfish skin tissues in our study. Among these, 49 were found to be located independently on different scaffolds, and 45 were in clusters containing two or more genes each, nearly twice that of *Fugu rubripes*. The number was 24 and 32, respectively [22]. A total of 139 claudin gene sequences have been reported in the goldfish genome based on the NCBI database (https://www.ncbi.nlm.nih.gov/gene/ (accessed on 19 August 2022)). Therefore, there must exist additional claudin transcripts in the other tissues of goldfish. The exact mechanisms of claudin evolution in goldfish, however, remain unknown. We speculated that the claudin multigene family expanded via gene duplication, just like in puffer fish. The prevalent tandem copies of claudins might be a result of gene replication. In contrast, we also speculated that the WGD event in evolution history might contribute partially to the amazingly high number of claudin genes. The evidence has indicated that the claudins are located on more than 31 chromosomes in our study. The located chromosome numbers of goldfish and zebrafish were 36 and 18, respectively (obtained from NCBI records). Indeed, some claudins were found to have no ortholog in mammals such as *cldnd* and *cldnj* [24], and the subfamily of some claudins, such as *cldn 4-like* and *cldn 8-like*, could be divided into three clades.

### 3.2. Cytoskeleton Reorganization and Vesicle Trafficking in Translocation of the Melanosome/Melanin

In the cyan goldfish, melanophores were found in the hypodermis and dermis, with the dermis having the most immature melanophores. In contrast, in the hypodermis, dermis, and epidermis of the black goldfish, most of the melanophores were darkened and thickened, with accumulated melanin, suggesting their mature stage and translocation of the melanosome [25]. Cytoskeleton organization-related proteins have been reported to play roles in the intracellular transport of melanosomes. The proteins catalyzing melanosome transport are also conserved in vertebrates [25]. Studies in mammalian melanophores and in related fish and frog melanophores revealed that microtubule and actin filament systems regulate the distribution of melanosomes with the companions of dynein, kinesin, and myosin V [26,27]. Early melanosomes are anchored on microtubules around the perinuclear area, driven by plus-end directed kinesins and minus-end directed dynesins [28]. This tug-of-war between kinesin and dynesin controls the ultimate direction of the melanosome transport [29]. During the transformation of goldfish color from cyan to black, the expression of kinesins and microtubule-associated proteins increased, and the melanosomes moved along the microtubule system from the nucleus toward the cell periphery. With the movement, the melanosomes become bigger and fully pigmented. The intracellular transport of melanosomes was accompanied by cytoskeleton reorganization in the dynamic transformation of goldfish color from cyan to white.

In the teleosts’ skin, the melanophores are mostly found in the dermis, although they have also been observed in the epidermis [30]. Our study suggested that melanophores could be found in the hypodermis, dermis, and epidermis of goldfish. In particular, dispersed melanosome/melanin was seen in the epidermal, DSS, and connectivity layer between the DSS and DSC, suggesting that these pigments could be secreted by melanophores and transferred extracellularly in skin tissues. This was not the sole report of extracellular melanosome/melanin in fish. Recently, large, rounded, and densely packed melanosomes have been reported to be loosely organized in the stroma of deep-sea fish species [31]. 

Though previous to this study little was known about the extracellular transfer of melanosome/melanin in fish, the intercellular melanosome/melanin movements in mammals and humans resulting from the interaction of melanophore dendritic tips with neighboring keratinocytes has drawn widespread attention [32,33]. The detailed mechanism of melanin transfer remains ambiguous. However, all current models recognize that vesicle trafficking is involved in melanosome/melanin transfer [34]. The evidence has also found that melanin translocation from melanophores is mediated by the small GTPase Rab11b [35] or depletion of Rab11b [36], Rab11a, Rab11b, and Rab17 [37], leading to impaired melanin transfer to keratinocytes. Rab11FIP proteins, interacting with MYO5B, participate in the spatially and temporally distinct steps of the recycling process for vesicles by interacting with Rab11 [38]. The different expressions of rab11 and rab11fib1, rab11fib4, and rab11fib5 in goldfish confirmed the extracellular transfer of melanin at the molecular level. During the intracellular transport of cargos, the soluble N-ethylmaleimide–sensitive factor attachment protein receptor (SNARE) protein complex is the most common fusogenic factor promoting membrane fusion. Syntaxins are a family of membrane proteins acting as target SNAREs, primarily localized to the plasma membrane [39,40,41]. VAMP8 is a vesicle SNARE involved in vesicle trafficking and motility control [42]. Here we found vamp8 and stx18, and at least six homologous genes of stx11 were expressed in skin tissues, and their expression was connected with the color transformation of goldfish. These results suggested that the melanosome/melanin could be translocated during the dynamic transformation of goldfish skin color, resulting in the decreased density of the pigment granules. The results were confirmed by the TEM data. The transport of melanophores and the extracellular transfer of melanosome/melanin might be therefore mediated by the SNARE complex and the Rab protein family, respectively.

### 3.3. Disappearance of Melanin in Color Transformation of Goldfish from Cyan to White

Melanophores are dendritic-shaped cells that extend their projections almost parallel to the plane of the skin [30,43], forming permanent structures [44]. We utilized cross- and parallel sectioning with Masson staining and TEM microscopy. However, neither melanophores nor melanosomes were observed in the white goldfish skin tissue. A possible reason was that the melanophores lost their melanosome/melanin granules, and the dendritic processes were emptied and collapsed in place rather than being drawn back into the cell body of the melanophores [45]. The large amounts of nuclei in the intercellular space of the adipocytes confirmed this speculation.

In humans, mammals, and birds, the melanosomes in epidermal melanophores are transported to the tips of the dendritic arbours for ultimate transfer to neighboring keratinocytes and are degraded as these keratinocytes move to the skin surface and differentiate [34]. Unlike birds and mammals, the mainstream is that the pigment cells of ecothermic vertebrates retain their pigments intracellularly rather than transferring them to keratinocytes. These melanin granules are visible throughout development [46,47]. Obviously, our results from Masson staining and TEM microscopy supported a different hypothesis. We thus proposed that melanin granules might be degraded intracellularly or transferred extracellularly from melanophores in goldfish. However, more evidence and further investigations are required to elucidate the answer to this question.

## 4. Materials and Methods

### 4.1. Sample Collection

Skin samples of 6-month-old goldfish *Carassius auratus* (Linnaeus, 1758) were collected from the F3 progenies of a hybrid family. In 2019, we built a hybrid family using red grass goldfish (♂) and cyan wakin goldfish (♀). In 2021, the F3 generation goldfish appeared cyan color in the whole body at the early development stage. The skin color of a certain amount of the goldfish transformed to black or white during development. Healthy CN, BK, and WH goldfish with similar length and weight were anesthetized using ethyl 3-aminobenzoate methanesulfonate (Sigma, Darmstadt, Germany, A5040). There were 8 replicates in each color group. Autopsy was carried out from the abdominal cavity. A 5 × 5 mm skin tissue piece above the lateral line was cut off just below the dorsal fin from each fish, removed the muscle tissues as much as possible, and rinsed thrice with PBS buffer.

### 4.2. Transcriptomics Sequencing and Analysis

The total RNA was extracted from skin tissues of cyan, black, and white color goldfish with Trizol reagent (Invitrogen, Thermofisher, Waltham, MA, USA, 15596026). A total of 3 ug RNA in each sample was used for the construction of a transcriptome sequencing library after an overall quality review using NEBNext^®^ Ultra™ RNA Library Prep Kit for Illumina^®^ (NEB, E7530L). Each library was diluted into 1ng/uL and tested for the insert size. All libraries were sequenced on the Illumina Novaseq S2 (PE150) platform for 150 bp paired-end reads.

Raw data were stored in FASTQ format. The adapters and reads with low quality or containing > 5% N bases were filtered out for clean data. The degenerate base symbol N stands for any nucleotide A/T/G/C (not a gap). The clean data were mapped to the reference genome of the goldfish (assembly ASM 336829v1) according to HISAT2 v2.1.0. The numbers of reads mapped to each gene were counted with HTSeq (http://www-huber.embl.de/users/an ders/HTSeq/doc/overview.html, 0.6.0 (accessed on 8 July 2022)) and calculated for expression level with the fragments per kilobase million mapped reads (FPKM) method.

The DEGs were analyzed from the cyan_vs_black goldfish group and the cyan_vs_ white goldfish group using DESeq2 (1.20.0) software, with |log_2_(Fold change)| ≥ 1 and q < 0.05 as the main parameters. Gene Ontology (GO, http://geneontology.org/ (accessed on 15 July 2022)) and the Kyoto Encyclopedia of Genes and Genomes (KEGG, http://www.kegg.jp/ (accessed on 15 July 2022)) enrichment analysis were conducted for gene function enrichment with q value (*p*.adjust) ≤ 0.05.

### 4.3. WGBS Analysis

Genomic DNA samples were extracted from the fish skin tissues. After qualification, the DNA samples were broken into 200 bp and modified for DNA methylation library construction. The libraries were sequenced on Illumina Novaseq S2 after passing the quality control. The raw data were obtained and stored in FASTQ format. To obtain clean data, adaptor-polluted reads, low-quality reads, and reads with over 10% N bases were removed by in-house script. Clean reads were mapped to the reference genome (assembly ASM 336829v1) by Bismark, and only uniquely mapped reads were retained. Cytosines were considered as methylated based on the binomial test followed by the Benjamini–Hochberg false discovery rate correction. The methylation level of a single cytosine was calculated as mC/(mC + umC), where the mC is the number of methylated reads and umC is the number of unmethylated reads. The methylation level of a region was defined as the mean methylation level of cytosines in that defined region.

DMRs were called by DSS software, q < 0.00005, FDR > 0.1. The DMRs included no less than three CpG sites. The genes related to the DMRs were then annotated using bedtools, and the GO term enrichment and KEGG pathway enrichment were based on the hypergeometric test with the threshold q < 0.05.

### 4.4. qPCR Validation of Selected Differently Expressed Genes

With the extracted RNA of the goldfish (CN, BK, and WH) as templates, the first strand of cDNA was synthesized via PrimeScript RT reagent Kit with gDNA Eraser (TaKaRa, RR047) based on the manufacture’s protocol. The qPCR primers of differently expressed genes *coro1c*, *sept2b*, *cldnda*, *nect2*, *myh*, and *col12a1* and reference gene *β2m*/*gapdh* were designed according to Primer3web (http://primer3.ut.ee/ (accessed on 29 July 2022)) (Supplementary Table S4). The reference genes were selected and evaluated according to the transcriptomic data. qPCR was conducted using Talent qPCRPreMix with SYBR GREEN I (Tiangen, Beijing, China, FP209) on an FastOne machine (Applied Biosystems, Thermofisher, Waltham, MA, USA). The program was performed as following: 95 °C 3 min; 40 cycles of 95 °C for 5 s; and 60 °C for 30 min. After the program melting curve analysis was conducted, the data were analyzed using the 2-^△△^Ctrelative quantification method with B2M/GAPDH as an inner control in triplicates. The significance was calculated by one-way ANOVA and a post hoc test with the SNK and Tukey methods, *p* < 0.01.

### 4.5. Histological Microscopy

A 5 × 5 mm skin piece above the lateral line was cut off just below the dorsal fin from the cyan, white, and black fish, separately. We removed the muscle tissues as much as possible and rinsed twice with PBS buffer. For Masson staining microscopy, the sample pieces were immediately transferred into 10% neutral buffered formalin (Sigma-Aldrich, HT5011) for tissue fixation preparation. The fixed skin tissues were washed with flushing water for paraffin section according to Liu’s method [48]. The embedded tissue waxes were cut in a thickness of 4 μm on LEICA RM2254. The slicers were dewaxed and stained with the Modified Masson’s Trichrome Stain Kit (Solarbio, Beijing, China, G1346) according to the manufacture’s protocol.

For TEM, the samples were immersed in 2.5% glutaraldehyde fixative solution (Solarbio, P1126) for 24 h, rinsed in 0.1 mol/L phosphate buffer solution (PH7.4) for 15 min in triplicate, then transferred into 1% Osmium tetroxide solution for 1 h. They were rinsed again in 0.1 mol/L phosphate buffer solution (PH7.4) for 15 min in triplicate. After dehydration in gradient concentrations of ethanol, the samples were treated with epoxy embedding medium (Sigma-Aldrich, Darmstadt, Germany, 45345) at 70 °C overnight. The embedded tissues were cut into 60–80 nm slices by the ultramicrotome (EM UC7, Leica, Weztlar, Germany). After staining with lead citrate and 2% bis (acetato-O)dioxouranium separately for 15 min, the skin slices were observed under the TEM system (Hitachi, Tokyo, Japan).

## 5. Conclusions

The present study constructed a hybrid family of goldfish to analyze the dynamic transformation of fish skin color, using red grass goldfish and cyan wakin goldfish as parents. When the color of the goldfish transformed from cyan to black or white, the melanosomes became pigmented and moved from the nucleus towards the cell periphery, motivated by the cytoskeleton reorganization. The expression of the cell junctions, especially the tight junction-related genes decreased, and thus, the cell–cell connectivity reduced. The melanosome/melanin was transferred out and moved further towards the dermal and epidermal layers with the vesicle trafficking system. The transcriptomic and DNA methylation analysis, accompanied by cellular data, suggested that it was the extracellular translocation rather than the biosynthesis or metabolism of the melanin process that resulted in the color transformation of the cyan goldfish. Our results will facilitate the understanding and the provide fundamental data for the breeding and improvement of cyan goldfish. However, further work is required for the gene function validation and regulation mechanism research.

## Data Availability

The datasets generated and/or analyzed during the present study are only available from the corresponding author on reasonable request.

## Supplementary Materials

The supporting information can be downloaded at: https://www.mdpi.com/article/10.3390/ijms232012214/s1.

## Abbreviations

BK: Black; C regions: Constant regions; C: Cytosine; CN: Cyan; DEGs: Differentially expressed genes; DMRs: Differentially methylated regions; DSC: Dermal stratum compactum; DSS: Dermal stratum spongiosum; EBM: Epidermal basement membrane; FPKM: Fragments Per Kilobase Million Mapped Reads; GO: Gene Ontology; HD: Hypodermis; KEGG: Kyoto Encyclopedia of Genes and Genomes; lncRNAs: Long noncoding RNAs; mCG: Methylated CGs; mCHG: Methylated CHGs; mCHH: Methylated CHHs; qPCR: Real-time quantitative PCR; SNARE: Soluble *N*-ethylmaleimide–sensitive factor attachment protein receptor; RNA-seq: Transcriptome sequencing; snoRNAs: Small nucleolar RNAs; snRNAs: Small nuclear RNAs; TEM: Transmission electron microscopy; V segments: Variable chains; WGBS: Whole genome bisulfate sequencing; WGD: Whole genome duplication; WH: White.
