# Supplementary figures and images for: Cell Junction and Vesicle Trafficking-Mediated Melanosome/Melanin Transfer Are Involved in the Dynamic Transformation of Goldfish Carassius auratus Skin Color
Source: Int J Mol Sci. 2022 Oct 13;23(20):12214. doi: 10.3390/ijms232012214 (PMC9603685; doi:10.3390/ijms232012214)

## Slide 1
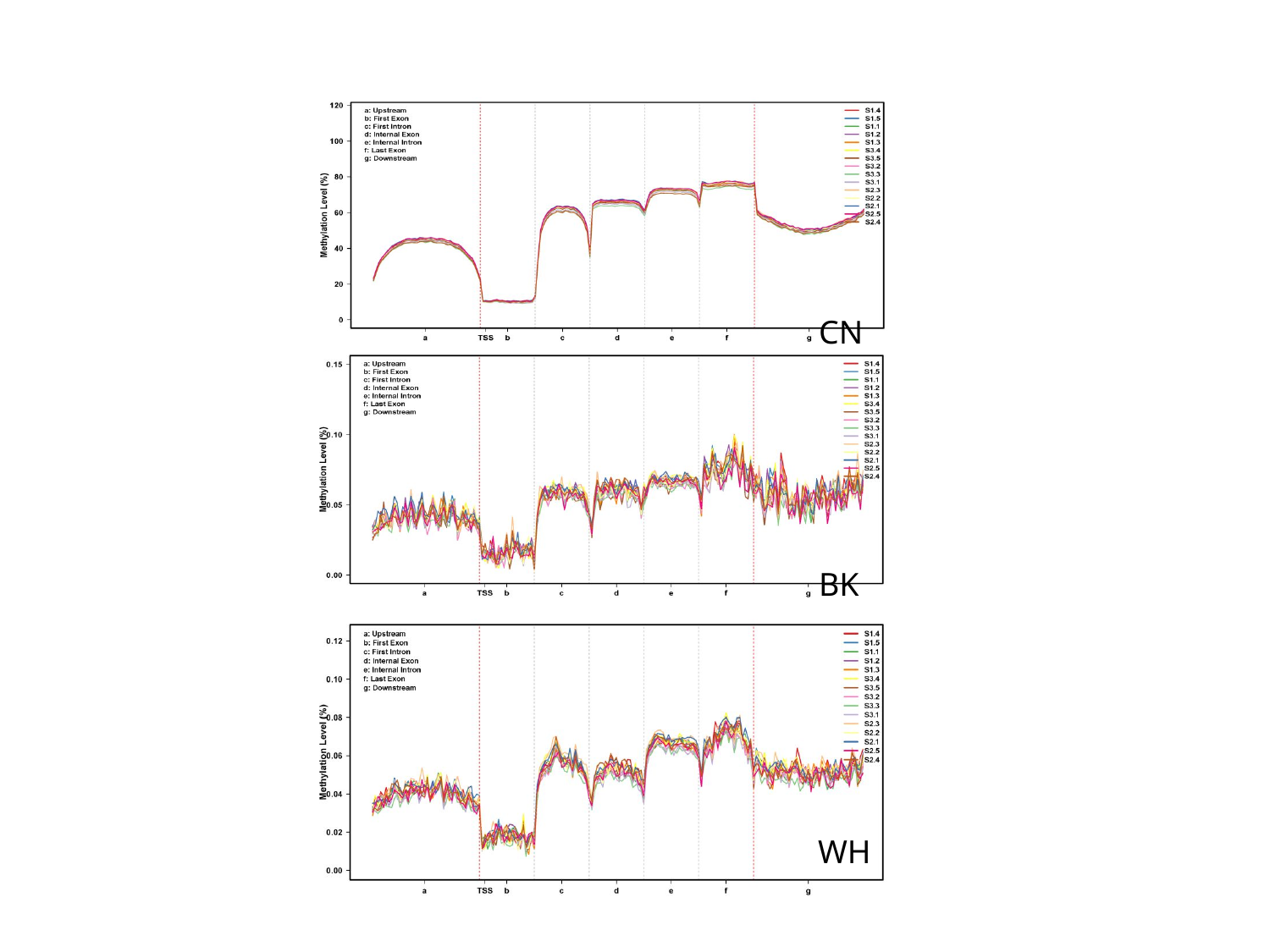

CN
BK
WH

## Slide 2
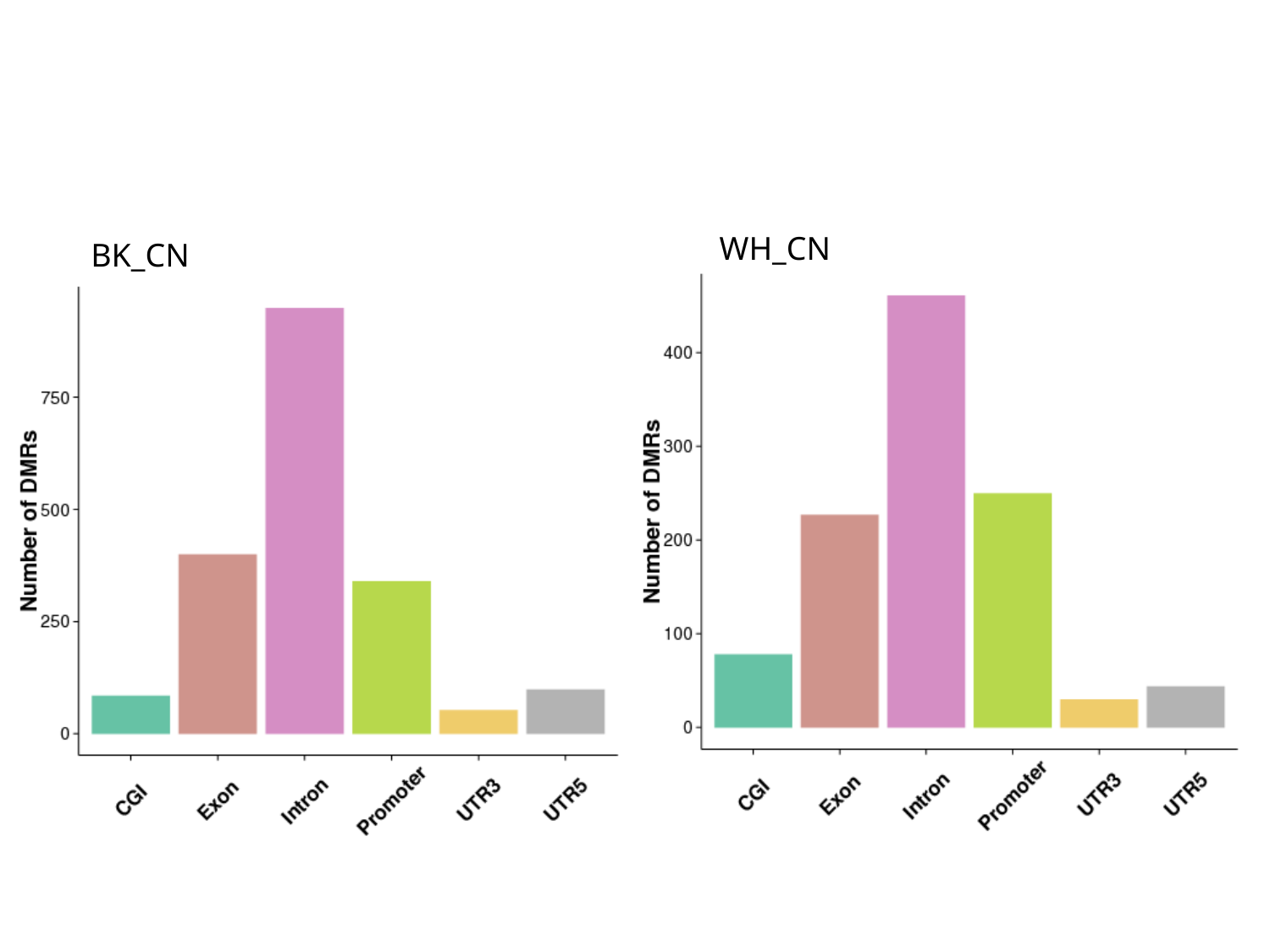

WH_CN
BK_CN

Supplement: Supplementary file 1 [file ijms-23-12214-s001.zip › Supplementary figures.pptx]
